# Effects of different forms of yeast *Saccharomyces cerevisiae* on growth performance, intestinal development, and systemic immunity in early-weaned piglets

**DOI:** 10.1186/s40104-015-0046-8

**Published:** 2015-11-14

**Authors:** Zongyong Jiang, Shaoyong Wei, Zhilin Wang, Cui Zhu, Shenglan Hu, Chuntian Zheng, Zhuang Chen, Youjun Hu, Li Wang, Xianyong Ma, Xuefen Yang

**Affiliations:** Key Laboratory of Animal Nutrition and Feed (South China), Ministry of Agriculture of China, State Key Laboratory of Livestock and Poultry Breeding, Guangdong Public Laboratory of Animal Breeding and Nutrition, Institute of Animal Science, Guangdong Academy of Agricultural Sciences, Guangzhou, 510640 China; Agro-biological Gene Research Center, Guangdong Academy of Agricultural Sciences, Guangzhou, 510640 China

**Keywords:** Growth performance, Immunity, Intestinal development, Piglets, *Saccharomyces cerevisiae*, Yeast

## Abstract

The present study was conducted to determine effects of different forms of yeast (*Saccharomyces cerevisiae*, strain Y200007) on the growth performance, intestinal development, and systemic immunity in early-weaned piglets. A total of 96 piglets (14-d old, initial average body weight of 4.5 kg) were assigned to 4 dietary treatments: (1) basal diet without yeast (Control); (2) basal diet supplemented with 3.00 g/kg live yeast (LY); (3) basal diet supplemented with 2.66 g/kg heat-killed whole yeast (HKY); and (4) basal diet supplemented with 3.00 g/kg superfine yeast powders (SFY). Diets and water were provided *ad libitum* to the piglets during 3-week experiment. Growth performance of piglets was measured weekly. Samples of blood and small intestine were collected at days 7 and 21 of experiment. Dietary supplementation with LY and SFY improved G:F of piglets at days 1-21 of the experiment (*P* < 0.05) compared to Control group. Serum concentrations of growth hormone (GH), triiodothyronine (T_3_), tetraiodothyronine (T_4_), and insulin growth factor 1 (IGF-1) in piglets at day 21 of the experiment were higher when fed diets supplemented with LY and SFY than those in Control group (*P* < 0.05). Compared to Control group, contents of serum urea nitrogen of piglets were reduced by the 3 yeast-supplemented diets (*P* < 0.05). Diets supplemented with LY increased villus height and villus-to-crypt ratio in duodenum and jejunum of piglets (*P* < 0.05) compared to other two groups at day 7 of the experiment. Feeding diets supplemented with LY and SFY increased (*P* < 0.05) serum concentrations of IgA, IL-2, and IL-6 levels in piglets compared to Control. The CD4^+^/CD8^+^ ratio and proliferation of T-lymphocytes in piglets fed diets supplemented with LY were increased compared to that of Control group at day 7 of the experiment (*P* < 0.05). In conclusion, dietary supplementation with both LY and SFY enhanced feed conversion, small intestinal development, and systemic immunity in early-weaned piglets, with better improvement in feed conversion by dietary supplementation with LY, while dietary supplementation with SFY was more effective in increasing systemic immune functions in early-weaned piglets.

## Introduction

Weaning is a critical time in current pig production systems. Weaning piglets have to undergo many challenges such as low feed intake, acute diarrhea and body weight loss, which are caused by nutritional, immunological and psychological disruptions [1, 2]. Nutritional strategies such as dietary supplementation of probiotics are used for improving intestinal development and immune function in weaned piglets [3, 4]. Yeast is one of the most commonly used probiotics in pig production. It has been reported that some strains (*Saccharomyces cerevisiae*, strain CNCM I-4407) and components of yeasts can improve growth performance and immune function in weaned piglets [5–7]. Dietary supplementation with live yeast had a positive effect on performance and health in weaned piglets through stimulating the immune system and maintaining a favorable intestinal environment [6]. Administration of live yeast *Saccharomyces cerevisiae* significantly increased the villus heights and IgA levels in the ileum [3, 4] and enhanced some immunological indices in the serum of piglets [7], and thus helped reduce postweaning diarrhea after enterotoxigenic *E. coli* infection. However, the effect of heat-killed yeast on growth performance, intestine development and immune function of weaned piglets remained unknown.

Moreover, previous studies have shown that the nucleotides extracted from yeast were shown to reduce occurrence of diarrhea in early-weaned pigs [8], while its cell wall products extracted from yeast were proved to improve the growth performance and health status of piglets [9]. However, it is difficult and expensive to extract the cell wall components from yeast [10]. Superfine grinding technology is a useful tool for preparing superfine powder with prominent quality in solubility, dispersion, adsorption, chemical reactivity and fluidity [11]. Studies have shown that superfine pulverization significantly enhanced the absorption efficiency of traditional Chinese medicine in rats [12]. Nevertheless, there are relatively few data regarding the comparison effects of live, heat-killed, and superfine yeasts on the intestinal development and postweaning systemic immunity in weaned piglets. Therefore, the purpose of this study was to evaluate how different forms of yeast supplementation influence the growth performance, development of the small intestine and systemic immunity in early-weaned piglets.

## Materials and methods

The experimental procedures performed in this study were approved by Animal Care and Use Committee at Guangdong Academy of Agricultural Sciences.

### Preparation of different forms of yeasts

The preparations of live yeast *Saccharomyces cerevisiae* (strain CCTCC Y200007) were obtained by high cell density fermentation in a 30 L-fermentor (B. Braun Biotech International GmbH, Melsungen, Germany). The fermentation liquor was subjected to freeze-drying to produce live yeast (LY) with viable yeast cells of 4.3 × 10^9^ CFU/g. Heat-killed yeast (HKY) was prepared by heating LY for 4 h at 105 °C. Superfine powder of yeast (SFY) was prepared from LY by using superfine grinding pulverizer (Jinan Billion Powder Technology Engineering Co., Ltd. China) at 4 °C and immediate screening through a 300 mesh sieve. Different forms of yeasts (LY, HKY, and SFY) were made from the same number of live yeast cells. Viable cells in LY, HKY, and SFY were detected by alkaline methylene blue staining [13] and examined with a Zeiss AxioScope A1 microscope (Carl Zeiss).

### Animals, diet, and management

Ninety-six *Duroc* × *Landrace* × *Yorkshire* piglets weaned at 14 d of age (initial average body weight at 4.5 kg) were randomly allotted to 4 treatments: (1) basal diet (Control), without yeast supplement (Table 1); (2) basal diet supplemented with 3.00 g/kg LY; (3) basal diet supplemented with 2.66 g/kg HKY; (4) basal diet supplemented with 3.00 g/kg SPY. There were 4 replicates (pens) for each treatment with 3 males and 3 females per replicate. The experiment lasted for 3 weeks. Body weight of piglet after overnight fasting and feed consumption per replicate was recorded at days 1, 7, 14, and 21 of the experiment to calculate the ADG, ADFI, and G: F accordingly.

**Table 1:** Composition and nutrients level of the basal diet

Ingredients	Percent	Calculated nutrients level	
Corn	52.51	CP, %	20.50
Soybean meal	18.00	ME, MJ/kg	14.42
Soy protein concentrate	4.50	Ca, %	0.88
Fish meal	8.00	Total P, %	0.70
Whey power	10.00	Lys, %	1.42
Soybean oil	3.50	Met, %	0.40
Dicalcium phosphate	0.60	Met + Cys, %	0.74
Limestone	0.68	Thr, %	0.95
L-Lysine · HCl	0.29	Trp, %	0.27
DL-Methioine	0.03		
L-Threonine	0.09		
Choline chloride	0.30		
Premix^a^	1.50		
Total	100.00		

^a^Provided per kg of basal diet: NaCl 1,500 mg, NaHCO_3_ 1,500 mg, Fe 330 mg, Cu 36 mg, Mn 240 mg, Zn 360 mg, Co 1.2 mg, Se 0.9 mg, I 2.1 mg, Vitamin A 20,000 IU, Vitamin D_3_ 2,800 IU, Vitamin E 30 mg, Vitamin K_3_ 5 mg, Vitamin B_1_ 3 mg, Vitamin B_2_ 10 mg, Vitamin B_6_ 8 mg, Vitamin B_12_ 40 mg, Niacin 40 mg, calcium pantothenate 15 mg, folic acid 1 mg, biotic 0.08 mg, bacitracin zinc 400 mg, 10 % colistin sulphate 500 mg, 10 % colistin sulfate 0.50 g

### Sample collections

At days 7 and 21 of the experiment, one piglet of each replicate (n = 4 per treatment) was randomly selected for blood samples collection via the anterior vena cava puncture after overnight fast. Serum samples were separated by centrifugation at 4 °C for 5 min (3,000× g) and stored at -20 °C until analysis. After collection of blood and injection of sodium pentobarbital (50 mg/kg BW, Sigma), one piglet (n = 4 per treatment) with similar BW to average pen weight was chosen from each replicate for sacrifice to collect intestinal tissues. Intestinal segments were defined as duodenum (to about 10 cm distal to the pylorus), jejunum (the middle portion of the small intestine), and ileum (from about 5 cm proximal to the ileocecal junction) according to the methods as previous described [14].

### Determinations of serum biochemical parameters

Concentrations of growth hormone (GH), triiodothyronine (T_3_), tetraiodothyronine(T_4_), insulin-like growth factors-1 (IGF-1), IL-2, and IL-6 in serum were determined by commercially available ELISA kits (Groundwork Biotechnology Diagnosticate, San Diego, CA). Serum urea nitrogen and IgA concentrations were determined with an automatic biochemical analyzer (CX-5, Beckman, Los Angeles, CA) following the instruction of the commercial kits from Beckman. Meanwhile, flow cytometry was used for detection of T lymphocyte subtype populations (CD4^+^/CD8^+^) in peripheral blood of piglets [15]. T-lymphocyte proliferation in peripheral blood of piglets at day 7 of the experiment were assessed using the 3-(4,5-dimethyl-2-thiazolyl)-2,5-diphenyl-2-H-tetrazolium bromide (MTT) assasy according to the method described previously [16].

### Small intestine morphology

Approximately 2-cm segments of the duodenum and jejunum at consistent locations were collected immediately, fixed in 10 % formalin, then subsequently embedded, sectioned and stained with hematoxylin and eosin by routine methods. Villus height, crypt depth, and villus-to-crypt ratio (V/C) of the small intestine were measured in approximately 10 microscopic fields using an image analysis system by a blinded investigator [17]. For determination of ultrastructure morphology of small intestine, the duodenum and jejunum samples at consistent locations were fixed in 2.5 % glutaric dialdehyde for 2 h and osmic acid for another 1 h. Samples were then dehydrated and embedded in resin, and cut intro ultrathin sections for staining with uranyl acetate. Samples were examined using a scanning electron microscopy (XL30ESEM) by a blinded investigator.

### Statistical analysis

Replicate (pen) was considered as the experimental unit for all the measurements for statistical analyses. Data were analyzed by one-way ANOVA via SPSS 13.0 software (SPSS Inc., Chicago, IL, USA). Results were presented as means with standard error (n = 4). Significant differences between groups were compared by Least-significant difference (LSD) for the post-hoc test. Differences were considered statistically significant at *P* < 0.05.

## Results

### Morphologies of different forms of yeast preparations

**Fig. 1:**
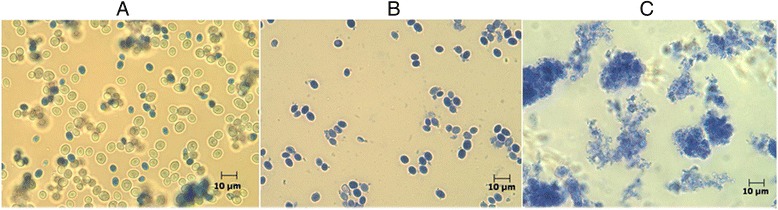
The morphologies of different forms of yeast preparations. Different forms of yeasts, including LY (**a**), HKY (**b**), and SFY (**c**) were resuspended and stained with methylene blue, respectively. The samples were examined with a Zeiss AxioScope A1 microscope (Carl Zeiss) at 400 × magnification under visible light. Abbreviations: LY, live yeast; HKY, heat-killed whole yeast; SFY, superfine yeast powders

By applying the staining of methylene staining, we found that most of yeast cells were live in LY (Fig. 1a) but few yeast cells were live in HKY (Fig. 1b). There were more than 99 % small fragments of yeast cells in SFY by ultrafine grinding (Fig. 1c), while less than 0.1 % viable yeast cells according to the plate counting result.

### Growth performance

During days 0 to 21 of the experiment, there was no significant difference in ADG and ADFI of piglets among dietary treatments (*P* > 0.05) (Table 2). However, dietary supplementation with LY improved the G: F (*P* > 0.05) of piglets at days 15-21 and days 0-21 relative to those fed basal diets and HKY-supplemented diets. There was no significant difference in G: F of piglets between SFY-supplemented group and other three groups (*P* > 0.05).

**Table 2:** Effects of different forms of yeasts *Saccharomyces cerevisiae* on growth performance of piglets^a^

Item	Dietary treatment^b^				SEM	*P* value
	Control	LY	HKY	SFY		
ADG, g/d						
Days 0- 7	60.88	71.68	64.27	67.80	5.20	0.52
Days 8-14	122.23	143.39	135.29	141.39	12.60	0.64
Days 15-21	200.64	218.77	230.71	216.54	10.80	0.32
Days 0-21	127.92	144.61	143.42	141.91	7.00	0.35
ADFI, g/d						
Days 0- 7	127.30	136.07	133.21	136.01	3.28	0.79
Days 8-14	231.04	234.71	246.21	240.43	8.15	0.94
Days 15-21	342.96	347.04	386.93	358.73	8.46	0.26
Days 0-21	233.77	239.27	255.45	245.06	5.40	0.58
G:F, g/d						
Day 0-7	0.48	0.53	0.48	0.50	0.02	0.27
Days 8-14	0.53	0.61	0.55	0.59	0.02	0.18
Days 15-21	0.59^d^	0.63^c^	0.60^d^	0.61^cd^	0.01	0.03
Days 0-21	0.55^d^	0.60^c^	0.56^d^	0.58^cd^	0.01	0.02

^a^Values are means for n = 4 replicates

^b^ *LY* live yeast, *HKY* heat-killed whole yeast, *SFY* superfine yeast powders

^c, d^Means in a row without a common letter differ (*P* < 0.05)

### Determinations of concentrations of serum urea nitrogen and hormones

As shown in Table 3, Serum urea nitrogen level of piglets was significantly reduced when fed the LY -supplemented and SFY-supplemented diets at both days 7 and 21 of the experiment in comparison to those in HKY-supplemented group (*P* < 0.001). Dietary supplementation with LY significantly increased serum concentrations of GH, T_3_, T_4_, and IGF-1 of piglets on day 21 of the experiment when compared with those in Control group (*P* < 0.05). Moreover, piglets fed diets supplemented with SPY had higher serum concentrations of GH, T3, and T4 than those in Control group (*P* < 0.05). Concentration of serum T_3_ and T_4_ in piglets from HKY-supplemented group were much higher than those from Control group on day 21 (*P* < 0.05).

**Table 3:** Effects of different forms of yeasts *Saccharomyces cerevisiae* on concentrations of serum urea nitrogen and hormones in piglets^a^

Item	Dietary treatment^b^				SEM	*P* value
	Control	LY	HKY	SFY		
Day 7						
GH, ng/mL	1.51	1.95	1.72	1.91	0.12	0.07
T_3_, ng/mL	0.86	1.20	1.10	1.12	0.09	0.10
T_4_, μg/L	63.30	78.88	73.65	73.98	3.81	0.08
IGF-1, ng/mL	460.08	504.64	466.40	487.59	11.9	0.08
Urea Nitrogen, mmol/L	5.66^c^	4.68^e^	5.17^d^	4.63^e^	0.15	0.001
Day 21						
GH, ng/mL	1.15^d^	2.01^c^	1.57^cd^	1.91^c^	0.14	0.004
T_3_, ng/mL	0.91^d^	1.49^c^	1.25^c^	1.26^c^	0.08	0.003
T_4_, μg/L	62.60^e^	86.50^c^	72.63^d^	78.05^d^	2.42	<0.001
IGF-1, ng/mL	456.63^e^	509.48^c^	465.45^de^	491.62^cd^	10.3	0.01
Urea Nitrogen, mmol/L	4.95^c^	4.02^e^	4.50^d^	4.05^e^	0.12	<0.001

^a^Values are means for n = 4 per replicates

^b^ *LY* live yeast, *HKY* heat-killed whole yeast, *SFY* superfine yeast powders

^c, d, e^Means in a row without a common letter differ (*P* < 0.05)

### Small intestinal morphology

**Fig. 2:**
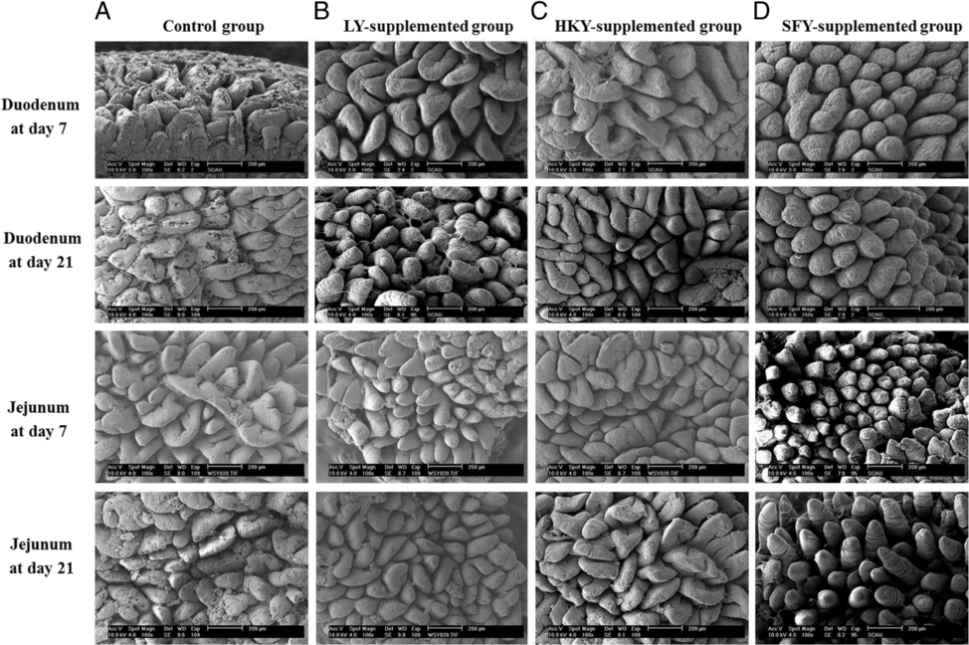
Analysis of intestinal ultrastructure morphology of piglets at days 7 and 21 by scanning electron microscopy. Duodenal and jejunal samples of piglets from (**a**) Control group, (**b**) LY-supplemented group, (**c**) HKY-supplemented group, and (**d**) SFY-supplemented group were examined with a scanning electron microscopy (XL30ESEM). Abbreviations: LY, live yeast; HKY, heat-killed whole yeast; SFY, superfine yeast powders

Villus height and villus-to-crypt ratio in both duodenum and jejunum were increased in piglets fed diets supplemented with LY or SFY when compared with those in Control group at days 7 and 21 of the experiment (*P* < 0.05) (Table 4 and Fig. 2). There were no differences in crypt depth of duodenum and jejunum in piglets, as well as villus height, crypt depth and villus-to-crypt ratio of ileum in piglets (data not shown) among dietary treatments at days 7 and 21 of the experiment (*P* > 0.05).

**Table 4:** Effects of different forms of yeasts *Saccharomyces cerevisiae* on the morphology of small intestine of piglets^a^

Item^b^	Dietary treatment^c^				SEM	*P* value
	Control	LY	HKY	SFY		
Duodenum						
Day 7 villus height, μm	358.91^f^	395.78^d^	367.64^ef^	382.25^de^	5.60	0.003
Day 7 crypt depth, μm	223.50	218.28	223.61	218.49	4.80	0.75
Day 7 V/C	1.61^e^	1.81^d^	1.64^e^	1.75^d^	0.03	<0.001
Day 21 villus height, μm	376.73	408.22	388.34	412.68	5.66	0.06
Day 21 crypt depth, μm	229.36	236.55	230.95	234.03	2.58	0.80
Day 21 V/C	1.64^f^	1.73^de^	1.68^ef^	1.76^d^	0.02	0.02
Jejunum						
Day 7 villus height, μm	361.80^e^	394.68^d^	368.09^e^	384.73^d^	4.90	0.002
Day 7 crypt depth, μm	221.81	218.28	219.43	215.29	3.60	0.65
Day 7 V/C	1.64^e^	1.81^d^	1.68^e^	1.79^d^	0.03	0.001
Day 21 villus height, μm	380.39	402.70	390.83	380.39	4.96	0.21
Day 21 crypt depth, μm	228.16	222.69	236.92	228.16	3.06	0.47
Day 21 V/C	1.67^ef^	1.81^d^	1.65^f^	1.67^ef^	0.03	0.04

^a^Values are means for n = 4 replicates. Diet LY contained 3 g/kg of live yeast; diet HKY contained 2.66 g/kg of heat-killed yeast; and diet SFY contained 3 g/kg of superfine yeast powder, both derived from LY

^b^V/C stands for villous height: crypt depth

^c^ *LY* live yeast, *HKY* heat-killed whole yeast, *SFY* superfine yeast powders

^d, e, f^Means in a row without a common letter differ (*P* < 0.05)

### Systemic immune parameters

Concentrations of IgA, IL-2 and IL-6 levels in the serum of piglets fed diets supplemented with LY or SPY were significantly increased compared to those in Control group at days 7 and 21 of the experiment (*P* < 0.05) (Table 5). There were no differences in serum concentrations of IgG, IgM, and TNF-α of piglets among dietary treatment at days 7 and 21 of the experiment (*P* > 0.05) (data not shown).

**Table 5:** Effects of different forms of yeasts *Saccharomyces cerevisiae* on concentrations of IgA, IL-2, and IL-6 in the serum of piglets^a^

Item	Dietary treatment^b^				SEM	*P* value
	Control	LY	HKY	SFY		
Day 7						
IgA, mg/L	46.66^d^	53.62^c^	51.41^cd^	54.49^c^	1.80	0.04
IL-2, pg/mL	33.98^d^	55.97^c^	39.39^d^	55.21^c^	2.80	<0.001
IL-6, pg/mL	16.91^d^	23.70^c^	18.98^d^	23.91^c^	1.10	0.001
Day 21						
IgA, mg/L	49.75^d^	58.25^c^	55.34^cd^	61.92^c^	2.20	0.01
IL-2, pg/mL	30.55^e^	66.02^c^	46.02^d^	67.09^c^	2.90	<0.001
IL-6, pg/mL	11.47^e^	25.15^c^	19.96^d^	24.53^c^	1.33	<0.001

^a^Values are means for n = 4 replicates

^b^ *LY* live yeast, *HKY* heat-killed whole yeast, *SFY* superfine yeast powders

^c, d, e^Means in a row without a common letter differ (*P* < 0.05)

The CD4^+^/CD8^+^ ratio of T lymphocytes at day 7 of the experiment was significantly higher in piglets fed the LY -supplemented diets than those in other groups (*P* = 0.044) (Table 6). T-lymphocyte proliferation rate (LPR) in piglets fed diets supplemented with LY or SFY was significantly increased on day 7 of the experiment compared with that in Control group (*P* = 0.038). However, there were no differences in percentage of CD4^+^ T and CD8^+^ T lymphocytes of piglets among groups at days 7 and 21 of the experiment (*P* > 0.05). Moreover, LPR of piglets at day 21 of the experiment did not differ among dietary treatment (*P* > 0.05).

**Table 6:** Effects of different forms of yeasts *Saccharomyces cerevisiae* on lymphocyte subtype populations and proliferation of T-lymphocytes of piglets at day 7^a^

Item^b^	Dietary treatment^c^				SEM	*P* value
	Control	LY	HKY	SFY		
CD_4_^+^ T, %	30.75	34.25	32.25	33.25	1.60	0.48
CD_8_^+^ T, %	25.25	22.50	24.75	23.25	1.00	0.20
CD_4_^+^ T/CD_8_^+^ T	1.22^e^	1.53^d^	1.31^de^	1.43^de^	0.07	0.04
LPR (OD_490_)	0.22^e^	0.33^d^	0.28^de^	0.31^d^	0.02	0.04

^a^Values are means for n = 4 replicates

^b^LPR stands for T-lymphocyte proliferation rate

^c^ *LY* live yeast, *HKY* heat-killed whole yeast, *SFY* superfine yeast powders

^d, e^Means in a row without a common letter differ (*P* < 0.05)

## Discussion

The present study was conducted to investigate the effect of different forms of yeast *Saccharomyces cerevisiae* on growth performance, intestinal development, and systemic immunity in early-weaned piglets. Our findings showed that the dietary supplementation with LY, but not HKY or SFY improved feed conversion ratio of early-weaned piglets. The enhancement of feed conversion ratio of piglets observed in our study was in agreement with previously published reports on live yeasts [4, 6, 18, 19].

Serum concentrations of hormones and urea nitrogen reflected the metabolic status of animals [20]. Insulin growth factors (IGFs) are integral components of multiple systems controlling both growth and metabolism [21] and serum urea nitrogen could reflect protein metabolism and amino acid balance of animals [22]. In the present study, dietary supplementation with LY and SFY increased serum concentrations of GH, T_3_, T_4_, and IGF-1, and reduced serum concentration of serum urea nitrogen in weaned piglets. These results indicated that dietary supplementation with LY and SFY could possibly increase rates of protein synthesis and lower rate of amino acid catabolism in weaned piglets. The positive effect of SPY may be due to the exposure of cell wall components of yeast which is a complex polymer and composed of β-glucans, α-mannans, mannoproteins and a minor component of chitin [23] after superfine grinding.

Weaning leads to villus atrophy due to the increase of apoptosis and the decrease of replacement of enterocytes within the crypts. Villus height and crypt depth were indirect indicators of the maturity and functional capacity of enterocytes, and longer villi provided an increased absorptive area in the small intestine [24]. Previous study has demonstrated that villus height was decreased in weaning transition with a consequently impairment of nutrient absorption [25]. Probiotics has been proved to contribute to the gut health of weaning piglets by enhancing the intestinal epithelial barrier [26]. In the present study, villus height and villus-to-crypt ratio in duodenum and jejunum were increased in piglets fed diets supplemented with LY and SFY. The improvement of small intestinal morphology in piglets fed yeast-supplemented diets is consistent with the previous studies [4, 19, 27]. These results indicated that both live yeast and its cell wall components could help enhance the small intestine development and physical intestinal epithelial barrier of early-weaned piglets. This effect could be due to the possible stimulation of yeast to enhance the polyamines release in piglets in intestinal lumen, which increased the proliferation rate of intestinal epithelial cells [28].

Many studies have demonstrated that supplementation with live yeast or polysaccharides from its cell walls may improve disease resistance and enhance performance through immunostimulation and maintenance of a favorable intestinal environment of animals [3, 9, 29, 30]. Previous study showed that when challenging with enterotoxinogenic *E. coli*, dietary supplementation with *Saccharomyces cerevisiae boulardii* increased intestinal IgA secretion and reduced bacterial translocation of piglets [3]. In the present study, dietary supplementation with LY and SFY increased serum IgA concentration in piglets than that in the Control group, which was in consistent with previous reports that live yeast (*Saccharomyces cerevisiae*, strain CNCM I-4407) increased piglet immunity during the postnatal period [5, 29]. Thus, the enhancement of systemic immunity by live yeast could possibly explain the improvement of growth performance of piglets when fed diets with supplementation of LY. Moreover, β-glucan from yeast with a thickness of ~115 nm [31], as a potent immunological activator [32–34], enhanced the functional activity of macrophages and activated antimicrobial activity of mononuclear cells and neutrophils [35, 36]. Dietary supplementation with β-glucans can modulate immune function and protect against pathogen infections [30, 37, 38]. Similarly, recent study demonstrated that yeast-derived microparticles (mainly cell walls) induced effective immune response against tumor cells *in vivo*, thus stimulating both humoral and cellular immunity [39]. Consistent with that, SFY used here was more effective than HKY in immunostimulation, possibly due to its smaller particle size of cell wall components in yeasts increasing the rate and extent of absorption or interaction with the enterocytes.

## Conclusions

Collectively, the present study demonstrated that dietary supplementation with LY or SFY improved feed conversion, promoted immune responses, and enhanced development of the small intestine in early-weaned piglets. Further studies are still warranted to illuminate the mechanism of how dietary supplementation with live yeast cells or the cell wall components modulate growth performance and immune function of early-weaned piglets.

## Abbreviations

BW: body weight
CP: crude protein
GH: growth hormone
HKY: heat-killed whole yeast
IGF-1: insulin growth factor 1
LY: live yeast
ME: metabolizable energy
SFY: superfine yeast powders
T_3_: triiodothyronine
T_4_: tetraiodothyronine
TNF-α: tumor necrosis factor
